# Comparison of reversal of rocuronium-induced neuromuscular blockade with sugammadex under remimazolam versus propofol anesthesia: a randomized clinical trial

**DOI:** 10.1007/s00540-025-03517-7

**Published:** 2025-05-26

**Authors:** Takashi Kawasaki, Masafumi Fujimoto, Naoyuki Hirata

**Affiliations:** 1 Department of Anesthesiology, Kumamoto University Hospital, 1-1-1, Honjyo, Cyuoku, Kumamoto-City, Kumamoto 860-8556 Japan; 2 Department of Anesthesiology, Kumamoto University School of Medical Sciences, Kumamoto University Hospital, 1-1-1, Honjyo, Cyuoku, Kumamoto-City, Kumamoto 860-8556 Japan

**Keywords:** Remimazolam, Sugammadex, Rocuronium

## Abstract

**Purpose:**

The anesthetic management combining remimazolam and neuromuscular blocking agents with sugammadex is expected to enhance the speed and safety of recovery from anesthesia. However, the effect of remimazolam on reversal of neuromuscular blockade with sugammadex remains unclear. We hypothesized that sugammadex could reverse rocuronium-induced neuromuscular blockade even under remimazolam anesthesia, although the recovery might be delayed. In the present study, the recovery time from rocuronium-induced neuromuscular blockade after administration of sugammadex under remimazolam anesthesia was compared with that under propofol anesthesia.

**Methods:**

Twenty-six patients over 18 years old scheduled for elective gynecological laparotomy under general anesthesia combined with epidural anesthesia were prospectively randomly assigned to remimazolam and propofol groups. After induction of general anesthesia with remifentanil and remimazolam or remifentanil and propofol, followed by their continuous infusion for anesthesia maintenance, train-of-four (TOF) responses were monitored following 0.9 mg/kg rocuronium administration. During surgery, rocuronium was infused continuously to maintain a TOF count of 1. At the end of surgery, when TOF counts of 2 were confirmed, sugammadex 2 mg/kg was administered and time to recovery of the TOF ratio to ≥ 90% of the baseline TOF ratio was compared between the two groups.

**Results:**

Median (interquartile range) recovery times in the remimazolam and propofol groups were 3.0 (2.3 to 3.8) and 2.5 (2.0 to 3.3) min, respectively (P = 0.62).

**Conclusion:**

Remimazolam anesthesia may not delay the efficacy of sugammadex in reversing rocuronium-induced neuromuscular blockade compared with propofol anesthesia.

**Clinical trial number and registry URL:**

The Japan Registry of Clinical trials (jRCT1071230073). URL: https://jrct.niph.go.jp/latest-detail/jRCT1071230073.

## Introduction

Sugammadex, a modified gamma-cyclodextrin, is the first selective relaxant-binding agent designed specifically to encapsulate aminosteroidal neuromuscular blocking agents, such as rocuronium or vecuronium. Formation of a complex of sugammadex and aminosteroidal neuromuscular blocking agents occurs at all levels of neuromuscular blockade (NMB), profound through shallow, and results in a rapid reversal of NMB [1].

Remimazolam is a newly developed benzodiazepine with a short duration of action [2]. Therefore, the anesthetic management combining remimazolam and neuromuscular blocking agents with sugammadex is expected to be used more frequently, because of its potential to enhance the speed and safety of recovery from anesthesia. However, the effect of remimazolam on NMB, including its reversal with sugammadex, remains unclear. Benzodiazepines are known to act as adenosine uptake inhibitors [3, 4], and midazolam, a benzodiazepine used in general anesthesia similarly to remimazolam, has been reported to potentiate the action of rocuronium by increasing endogenous adenosine concentration in an in vitro study [5]. Therefore, the efficacy of sugammadex may be indirectly reduced by benzodiazepines. However, the interaction between sugammadex and benzodiazepines other than remimazolam also remains poorly understood due to their limited utilization in anesthetic practice, making it challenging to predict the effect of remimazolam on reversal of rocuronium-induced NMB with sugammadex.

The purpose of this study was to clarify the effect of remimazolam on reversal of rocuronium-induced NMB with sugammadex. It is known that reversal of rocuronium-induced NMB with sugammadex is effective under sevoflurane anesthesia, which is known to enhance the effect of rocuronium [6–9]. Therefore, we hypothesized that sugammadex could reverse rocuronium-induced neuromuscular blockade even under remimazolam anesthesia, although the recovery might be delayed. To test this hypothesis, we investigated the recovery time from moderate NMB after administration of sugammadex under remimazolam anesthesia, and compared it with that under propofol anesthesia. In addition, we also compared the time-course of rocuronium action under remimazolam anesthesia with that under propofol anesthesia.

## Methods

### Ethical considerations

This prospective randomized clinical trial was conducted according to the recommendations of the Helsinki declaration. The trial was approved by the ethics committee of Kumamoto University Hospital (protocol Sennshinn-2562; October 3, 2023) and prior written informed consent was obtained from all subjects participating in the trial. The trial was registered prior to patient enrollment in the Japan Registry of Clinical trials (jRCT1071230073, October 12, 2023; Principal investigator: Masafumi Fujimoto). This manuscript adheres to the applicable CONSORT guidelines.

### Inclusion and exclusion criteria

Patients over 18 years old scheduled for elective gynecological laparotomy of an estimated 4–8 h duration, under general anesthesia combined with epidural anesthesia in the supine position, were assessed for eligibility to participate. All patients were American Society of Anesthesiologists physical status class I or II. Patients contraindicated for receiving benzodiazepines or propofol, and those with neuromuscular disorders, cirrhosis, hepatitis, cholestasis, heart failure, renal dysfunction and obesity (body mass index ≥ 30.0 kg/m^2^) were excluded from the study.

### Randomization and blinding

The chief investigator (M.F.) enrolled participants, and those found eligible for this study were randomly assigned to remimazolam or propofol groups according to the random allocation sequence generated by a computer program (Excel™, Microsoft, Redmond, Washington, USA). Randomization, including generation of the random allocation sequence, was conducted by an anesthesiologist who was not involved in this study. The attending anesthesiologist was informed about the allocation after obtaining written informed consent from each patient, but patients were blinded to group assignments.

### Anesthetic management

We followed the methods described by Fujimoto et al. in 2022 [10]. None of the patients received premedication. After the patients entered the operation room, routine monitoring, including of the electrocardiogram, pulse oximetry, non-invasive blood pressure, end-tidal CO_2_ measurement and anesthetic depth monitoring (BISx module NK™, Nihon Kohden, Tokyo, Japan) was applied. An intravenous cannula was inserted into the forearm vein for routine anesthetic and study drug administration. In all patients, before the induction of general anesthesia, an epidural catheter was inserted through the T11/12 interspace. One minute after a continuous infusion of remifentanil 0.25–0.50 μg/kg/min was commenced, patients in the remimazolam group received a continuous injection of remimazolam at 12 mg/kg/h (loading dose) until loss of the eyelash reflex according to the drug manufacturer’s recommendations, followed by a continuous infusion of remimazolam at 0.6–1.5 mg/kg^/^h. In the propofol group, general anesthesia was induced with a continuous infusion of remifentanil and a bolus injection of propofol 1.5–2.0 mg/kg, followed by a continuous infusion of propofol 4.0–7.0 mg/kg/h. The infusion rates of remimazolam and propofol were adjusted to maintain BIS values between 40 and 60. The continuous remifentanil infusion was adjusted between 0.1–0.5 μg/kg/min to provide optimal patient care, based on maintenance of blood pressure within 20% of baseline values. Before the start of the operation, 3–5 ml of 0.2% levobupivacaine was administered as a bolus into the epidural space, followed by its continuous epidural administration at the rate of 3–5 ml/h.

### Neuromuscular monitoring

After anesthesia induction, mask ventilation was performed and neuromuscular monitoring was commenced with an electromyograph and display unit (AF-201P™ and VA-201R™, Nihon Kohden, Tokyo, Japan). All neuromuscular monitoring was conducted in accordance with the guidelines established by the Good Clinical Research Practice in pharmacodynamic studies of neuromuscular blocking drugs [11]. After cleansing the skin with alcohol wipes, single-use surface electrodes for AF-201P (NM-345Y™, Nihon Kohden) were placed to stimulate the ulnar nerve on the arm opposite to that with the blood pressure cuff, with the sensing electrode being placed on the abductor digiti minimi (ADM) muscle, according to the manufacturer’s instructions. Neuromuscular data of the ADM muscle were transferred online to a computer and recorded. The patient’s study arm was immobilized and supramaximal stimulation was automatically ensured by the built-in calibration function. The detection threshold was manually configured to be 5% of the control value of the compound muscle action potential.

After confirmation of stable responses to train-of-four (TOF) stimulation and recording of the baseline TOF ratio, a single dose of 0.9 mg/kg rocuronium was administered according to actual body weight, followed by TOF stimulation every 15 s. Immediately after disappearance of the TOF responses, tracheal intubation was performed and the TOF monitoring interval was changed to every 1 min. Next, after reappearance of the 1st (T1) and 2nd (T2) twitch responses to TOF stimulation and spontaneous recovery of the T1 response to 25% of the control value were documented, a continuous infusion of rocuronium of 3–10 μg/kg/min was commenced to maintain a TOF count of 1. The rocuronium infusion rate was adjusted at the discretion of the attending anesthesiologist, to maintain the T1 response between 10 and 15% of the control value, and temporary suspension and bolus rocuronium administration of 0.05 mg/kg were permitted as many times as needed. Infusion of rocuronium was discontinued at the end of surgery, and TOF monitoring every 15 s was started after reappearance of the T2 response. When three continuous TOF counts of 2 were confirmed, 2 mg/kg sugammadex was administered. Once the TOF ratio had recovered to ≥ 90% of the baseline TOF ratio, administration of anesthetic agents was discontinued. Additionally, in the remimazolam group, flumazenil was administered at the discretion of the attending anesthesiologist. Patients were extubated after observation of sufficient recovery of consciousness and spontaneous respiration (≥ 8 breaths/min). Epidural infusion of 0.2% levobupivacaine was continued for postoperative analgesia.

During neuromuscular monitoring, esophageal temperature was measured as the central body temperature and maintained at ≥ 35 °C. Additionally, peripheral body temperature was measured continuously with a thermistor at the thenar eminence of the palm, and maintained at ≥ 32 °C with a forced air warming device.

### Statistical analysis

As the primary outcome, recovery time, defined as the time from administration of sugammadex to recovery of the TOF ratio to ≥ 90% of the baseline value was compared between patients receiving maintenance anesthesia with remimazolam and those receiving propofol. Reportedly, in patients receiving maintenance anesthesia with propofol, the average recovery time and its standard deviation (SD) with a single 2 mg/kg dose of sugammadex at the time of reappearance of the T2 response is 1.8 ± 0.7 min [6]. Based on our clinical practice, we considered a delay of less than 1 min in recovery time as being clinically insignificant. In the present study, therefore, a 50% delay, of approximately 1 min, was defined as a significant change, and the sample size was calculated to detect this delay with a SD of 0.7. Since a prior power analysis indicated that the sample size required in the present study, with a risk of type I error of α = 0.05 and type II error of β = 0.20, was 10 in each group, we decided to recruit 15 patients in each group to adjust for missing data.

The secondary outcomes of the present study were the time from initial administration of rocuronium to disappearance of the TOF response (onset time), time to reappearance of T1 and T2 responses, and time to spontaneous recovery of the T1 response to 25% of the control value (duration time).

Continuous variables were tested for normality using the Shapiro–Wilk test. Parametric data were expressed as the mean ± SD and compared using the t-test, while nonparametric data were expressed as the median (interquartile range) and compared using the Mann–Whitney *U* test. All statistical analyses, including the sample size calculation, were performed with the R statistical package version 3.4.1 (R Foundation for Statistical Computing, Vienna, Austria), and a p-value of < 0.05 was considered statistically significant.

## Results

**Fig. 1:**
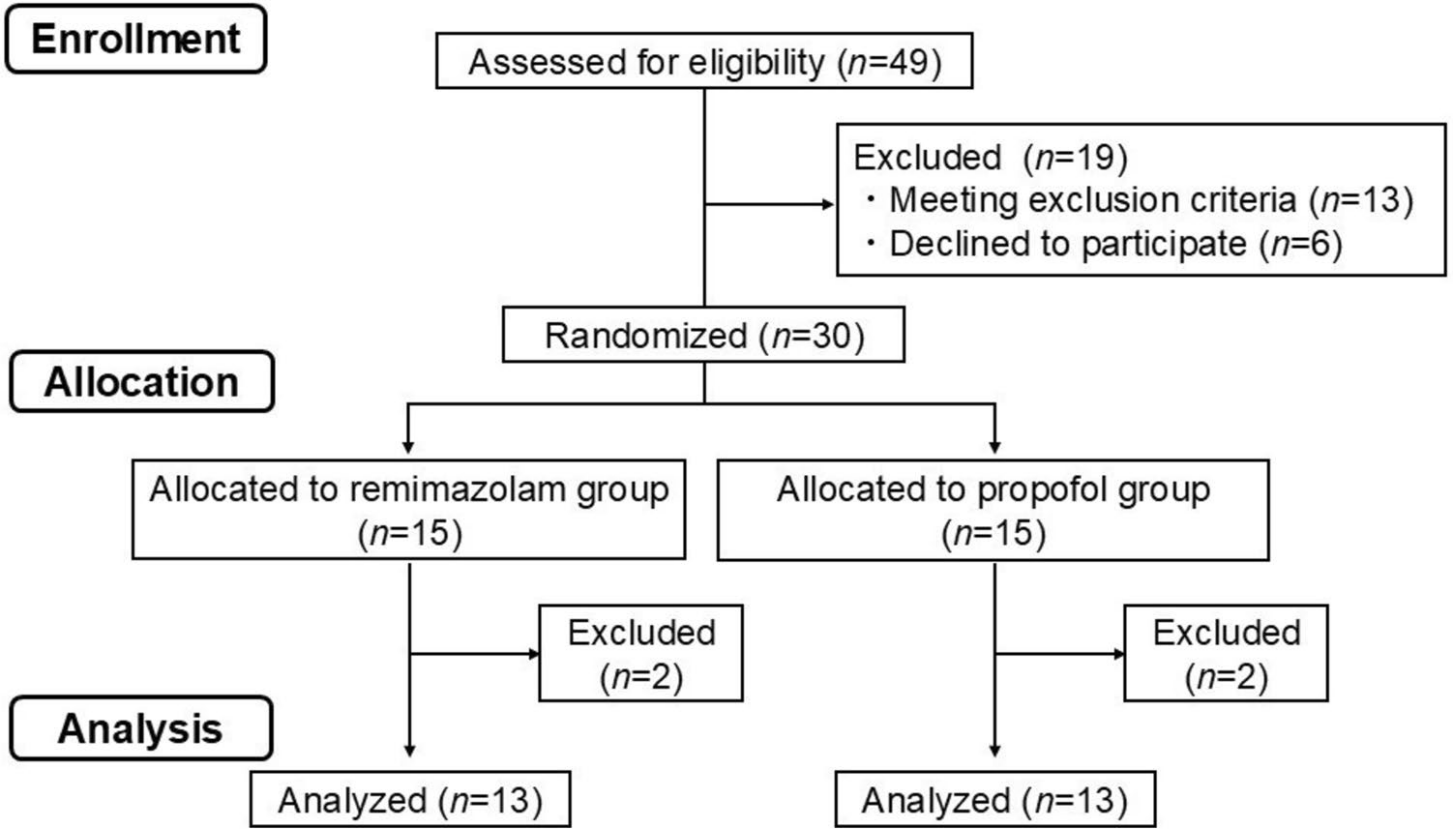
Flow diagram of study participation

The data were collected at Kumamoto University Hospital from October 31, 2023, to August 15, 2024. During this period, 49 patients were assessed for study eligibility, 19 of whom were excluded. One of the remaining 30 patients, assigned to the remimazolam group, was excluded because of the combined administration of propofol during surgery due to the persistence of a high BIS value. Another patient assigned to the remimazolam group was also excluded because the initial rocuronium dose was accidentally administered prior to calibration of the neuromuscular monitoring device. In addition, two patients assigned to the propofol group were excluded due to unstable neuromuscular monitoring. Therefore, a total of 26 patients (remimazolam group: 13, propofol group: 13) were included in the final analysis (Fig. 1). The patients’ basic demographic data and neuromuscular monitoring data before rocuronium administration are shown in Tables 1 and 2, respectively.

**Table 1:** Demographic data of the patients

	Remimazolam group (*n* = 13)	Propofol group (*n* = 13)	*P* value
Age (year)	51.7 ± 14.0	57.2 ± 12.2	0.30
Height (cm)	157.4 ± 6.4	156.3 ± 8.0	0.71
Weight (kg)	57.3 ± 5.7	58.0 ± 8.3	0.79
Body mass index (kg/m^2^)	23.2 ± 2.8	23.5 ± 3.5	0.62

Data were expressed as the mean ± standard deviation

**Table 2:** Neuromuscular monitoring data before rocuronium administration

	Remimazolam group (*n* = 13)	Propofol group (*n* = 13)	*P* value
Electrical stimulation current (mA)	27.9 ± 7.2	29.3 ± 5.8	0.59
Control value of the compound muscle action potential (mV)	11.1 ± 2.4	10.9 ± 2.7	0.81
Baseline TOF ratio	1.02 ± 0.01	1.01 ± 0.01	0.76

Data were expressed as the mean ± standard deviation

TOF, train-of-four

**Fig. 2:**
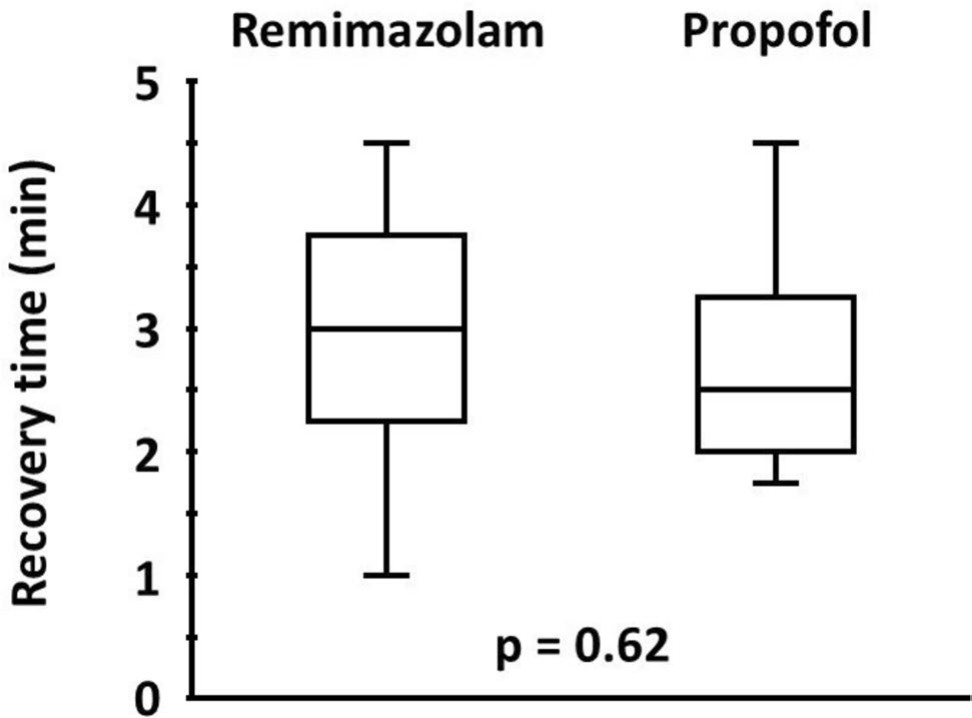
Recovery times in the two groups. Between remimazolam and propofol groups, there were no significant differences in the recovery time defined as the time from administration of sugammadex 2 mg/kg at the reappearance of T2 response to recovery of the TOF ratio to ≥ 90% of the baseline TOF ratio (P = 0.62). The *solid lines* represent the medians, and the *boxes* represent the interquartile range (median ± 25%). The *upper whiskers* indicate the maximum observations. The *lower whiskers* indicate the minimum observations. T2 response, 2nd twitch response to train-of-four stimulation; TOF, train-of-four

There were no significant differences in neuromuscular conditions at the administration of sugammadex (Table 3), nor were there differences in recovery time (min) between the remimazolam and propofol groups [3.0 (2.3 to 3.8) vs. 2.5 (2.0 to 3.3), P = 0.62] (Fig. 2).

**Table 3:** Neuromuscular conditions at the administration of sugammadex

	Remimazolam group (*n* = 13)	Propofol group (*n* = 13)	*P* value
Additional dose of rocuronium (μg/kg/min)	5.9 (4.1 to 6.8)	4.5 (4.2 to 6.6)	0.55
T1 response (%)	18 (15 to 23)	20 (18 to 23)	0.70
T2 response (%)	5 (5 to 6)	5 (5 to 6)	0.58

Additional dose of rocuronium was calculated as (additional dose of rocuronium)/(patient’s body weight)/(duration from the start of continuous infusion of rocuronium to sugammadex administration)

Data were expressed as the median (interquartile range)

T1 response, 1st twitch response to train-of-four stimulation; T2 response, 2nd twitch response to train-of-four stimulation

There were no requests from the surgeons for the administration of neuromuscular blocking agents during the operation, and data on T1 reappearance time, T2 reappearance time, and duration time were successfully collected from all patients. The results in relation to these secondary outcomes of the study are shown in Table 4. Neither onset time, T1 reappearance time, T2 reappearance time nor duration time of rocuronium were significantly different between the remimazolam and propofol groups.

**Table 4:** Results of secondary outcomes of the study

	Remimazolam group (*n* = 13)	Propofol group (*n* = 13)	*P* value
Onset time (min)	1.8 (1.5 to 2.0)	1.8 (1.3 to 1.8)	0.14
T1 reappearance time (min)	45 (38 to 53)	55 (44 to 57)	0.13
T2 reappearance time (min)	55 (49 to 64)	69 (55 to 71)	0.14
Duration time (min)	57 (51 to 69)	71 (61 to 75)	0.13

Data were expressed as the median (interquartile range)

T1, 1st twitch response to train-of-four stimulation; T2, 2nd twitch response to train-of-four stimulation

There were no complications or adverse events related to the study during the surgery or in the postoperative period in either group.

## Discussion

To the best of our knowledge, the present study is the first to investigate the effect of remimazolam on reversal of rocuronium-induced NMB with sugammadex. However, as no statistically significant difference on the recovery time was observed between the groups, definitive conclusions could not be drawn from the present study.

Benzodiazepines (e.g., diazepam, midazolam) are known to have centrally acting muscle relaxant effects that are induced by reduction of polysynaptic reflexes in the spine, which are thought to relate to the potentiation of some gamma-aminobutyric acid (GABA)-ergic inhibitory processes [12]. Enhancement of the inhibitory action of GABA-ergic cortical interneurons, which decreases the excitability of pyramidal cells and reduces the number of neurons available for generation of descending pyramidal tract activity, is also considered one of the factors contributing to the centrally acting muscle relaxant effects [13]. While remimazolam is expected to have centrally acting muscle relaxant effects similar to other benzodiazepines, it has already been reported that remimazolam does not affect the TOF ratio, suggesting the absence of any influence on the neuromuscular junction [14]. Absence of a significant difference in baseline TOF ratios between the two groups in the present study was consistent with the previous report. On the other hand, sugammadex acts by removing free rocuronium molecules from plasma and creating a concentration gradient favoring the diffusion of rocuronium away from the neuromuscular junction back into the plasma, where it is encapsulated by free sugammadex molecules, resulting in rapid reversal of NMB [15–17]. Therefore, it is considered that remimazolam does not directly affect reversal of NMB with sugammadex. Additionally, as there was no significant difference in the compound muscle action potential immediately after loading dose of remimazolam, compared to the propofol group, remimazolam may not have a direct effect on muscle contraction.

The present study also indicated that neither onset time, T1 reappearance time, T2 reappearance time nor duration of rocuronium action were significantly different between remimazolam and propofol anesthesia. Several reports have investigated the interaction of other benzodiazepines with neuromuscular blocking agents [5, 18, 19]. Olkkola et al. reported that the infusion rate of rocuronium necessary to produce a constant 90% block in T1 response relative to the control value under midazolam anesthesia did not differ significantly from that under propofol anesthesia [18]. On the other hand, according to Driessen et al., recovery of the twitch response after administration of vecuronium and atracurium under midazolam anesthesia was significantly delayed compared with those under diazepam anesthesia [19]. Moreover, supratherapeutic concentration of midazolam has been reported to potentiate the action of rocuronium by increasing endogenous adenosine concentration [5]. Considering these reports, the interaction between each benzodiazepine, including remimazolam, and neuromuscular blocking agents appears to vary not only with its type but also with the dose administered. As the remimazolam dose in the present study was adjusted according to routine clinical practice (i.e., BIS value), its dose-dependency remains unclear. Since a temporary decrease in motor-evoked potentials has been reported after switching anesthesia from propofol to remimazolam using a loading dose [20], the lack of significant differences between the groups might be explained by the relatively low dose of remimazolam used in the present study. Early recovery from anesthesia, which is one of the specific advantages of remimazolam [2], is valuable, particularly in emergency situations, such as “cannot ventilate, cannot intubate”. From this perspective as well, the effect of a high dose of remimazolam on reversal of NMB with sugammadex needs to be clarified in the future.

In addition, this study has several other limitations. First, neuromuscular monitoring was performed at the ADM muscle with the electromyograph. In the present study, sugammadex 2 mg/kg was administered at a TOF count of 2 according to the recommendations of the drug manufacturer, which was, however, decided based on studies that monitored neuromuscular function of the adductor pollicis (AP) muscle by acceleromyography [21–23]. Reportedly, electromyography is known to frequently indicate recovery later than that shown by acceleromyography [24]. It has also been reported that the TOF count of the AP muscle measured using acceleromyography was almost 4 when that of the ADM muscle measured using electromyography was 2 [25]. According to Pongrácz et al., 1 mg/kg sugammadex rapidly and effectively reverses rocuronium-induced NMB when the TOF count of the AP muscle measured with the acceleromyograph is 4 [26]. Thus, studies using acceleromyography and different doses of sugammadex are also needed to confirm our results. However, since the recommended dose of sugammadex is not differentiated based on the neuromuscular monitoring device, this study holds significant clinical relevance.

Second, the attending anesthesiologists could not be blinded to group assignments in this study, since it directly impacted anesthesia management. Although there might have been bias in adjustment of the rocuronium infusion rate during the operations in the present study, neuromuscular conditions at the administration of sugammadex (T1 and T2 values) were standardized between the groups. Therefore, the results of the primary observation were not likely to have been influenced in this study.

Finally, the sample size, which was determined to detect a delay of approximately 1 min in recovery time, was small, and the results are limited to middle-aged female patients. A much larger clinical trial (e.g., a non-inferiority trial) is required to conclusively demonstrate that reversal of rocuronium-induced NMB with sugammadex is equally effective under remimazolam and propofol anesthesia. However, the results of the present study may provide useful information for clinical practice.

In conclusion, we compared the effects of remimazolam on reversal of rocuronium-induced NMB with sugammadex versus that of propofol. In routine anesthetic practice, remimazolam may not delay the recovery from moderate NMB following sugammadex administration. However, it is impossible to draw any conclusion from the present study. 

## Data Availability

The datasets are available from the corresponding author on reasonable request.

## References

[1] Brull SJ, Meistelman C, Nemes R, de Boer HD, Thilen SR, Eriksson LI. Pharmacology of neuromuscular blocking drugs and antagonists (reversal agents). In: Gropper MA, Cohen NH, Eriksson LI, Fleisher LA, Johnson-Akeju S, Leslie K, editors. Miller’s anesthesia. 10th ed. Philadelphia: Elsevier Inc; 2025. p. 727–37.

[2] Kilpatrick GJ, McIntyre MS, Cox RF, Stafford JA, Pacofsky GJ, Lovell GG, Wiard RP, Feldman PL, Collins H, Waszczak BL, Tilbrook GS. CNS 7056: a novel ultra-short-acting Benzodiazepine. Anesthesiology. 2007;107:60–6. 17585216 10.1097/01.anes.0000267503.85085.c0

[3] Hammond JR, Paterson AR, Clanachan AS. Benzodiazepine inhibition of site-specific binding of nitrobenzylthioinosine, an inhibitor of adenosine transport. Life Sci. 1981;29:2207–14. 6119591 10.1016/0024-3205(81)90492-6

[4] Bender AS, Hertz L. Similarities of adenosine uptake systems in astrocytes and neurons in primary cultures. Neurochem Res. 1986;11:1507–24. 2891057 10.1007/BF00965770

[5] Narimatsu E, Niiya T, Kawamata M, Namiki A. Adenosine and adenosine uptake inhibitors potentiate the neuromuscular blocking action of rocuronium mediated by adenosine A(1) receptors in isolated rat diaphragms. Acta Anaesthesiol Scand. 2008;52:1415–22. 19025536 10.1111/j.1399-6576.2008.01714.x

[6] Vanacker BF, Vermeyen KM, Struys MM, Rietbergen H, Vandermeersch E, Saldien V, Kalmar AF, Prins ME. Reversal of rocuronium-induced neuromuscular block with the novel drug sugammadex is equally effective under maintenance anesthesia with propofol or sevoflurane. Anesth Analg. 2007;104:563–8. 17312209 10.1213/01.ane.0000231829.29177.8e

[7] Rex C, Wagner S, Spies C, Scholz J, Rietbergen H, Heeringa M, Wulf H. Reversal of neuromuscular blockade by sugammadex after continuous infusion of rocuronium in patients randomized to sevoflurane or propofol maintenance anesthesia. Anesthesiology. 2009;111:30–5. 19512873 10.1097/ALN.0b013e3181a51cb0

[8] Lowry DW, Mirakhur RK, McCarthy GJ, Carroll MT, McCourt KC. Neuromuscular effects of rocuronium during sevoflurane, isoflurane, and intravenous anesthesia. Anesth Analg. 1998;87:936–40. 9768798 10.1097/00000539-199810000-00036

[9] Wulf H, Ledowski T, Linstedt U, Proppe D, Sitzlack D. Neuromuscular blocking effects of rocuronium during desflurane, isoflurane, and sevoflurane anaesthesia. Can J Anaesth. 1998;45:526–32. 9669005 10.1007/BF03012702

[10] Fujimoto M, Kubota F, Kaneda H. Effects of neuromuscular blockade on the surgical conditions of laparoscopic totally extraperitoneal inguinal hernia repair: a randomized clinical trial. Hernia. 2022;26:1179–86. 35107670 10.1007/s10029-022-02570-5

[11] Fuchs-Buder T, Brull SJ, Fagerlund MJ, Renew JR, Cammu G, Murphy GS, Warlé M, Vested M, Fülesdi B, Nemes R, Columb MO, Damian D, Davis PJ, Iwasaki H, Eriksson LI. Good clinical research practice (GCRP) in pharmacodynamic studies of neuromuscular blocking agents III: the 2023 Geneva revision. Acta Anaesthesiol Scand. 2023;67:994–1017. 37345870 10.1111/aas.14279

[12] Farkas S, Tarnawa I, Berzsenyi P. Effects of some centrally acting muscle relaxants on spinal root potentials: a comparative study. Neuropharmacology. 1989;28:161–73. 2716970 10.1016/0028-3908(89)90053-1

[13] Schönle PW, Isenberg C, Crozier TA, Dressler D, Machetanz J, Conrad B. Changes of transcranially evoked motor responses in man by midazolam, a short acting benzodiazepine. Neurosci Lett. 1989;101:321–4. 2771175 10.1016/0304-3940(89)90553-3

[14] Yuruki T, Fujimoto M, Hirata N. The effect of remimazolam on the baseline TOF ratio: a prospective, clinical study. Anesthesiol Res Pract. 2024. 10.1155/anrp/9990140. 39640500 10.1155/anrp/9990140 PMC11620802

[15] Naguib M. Sugammadex: another milestone in clinical neuromuscular pharmacology. Anesth Analg. 2007;104:575–81. 17312211 10.1213/01.ane.0000244594.63318.fc

[16] Gijsenbergh F, Ramael S, Houwing N, van Iersel T. First human exposure of Org 25969, a novel agent to reverse the action of rocuronium bromide. Anesthesiology. 2005;103:695–703. 16192761 10.1097/00000542-200510000-00007

[17] Epemolu O, Bom A, Hope F, Mason R. Reversal of neuromuscular blockade and simultaneous increase in plasma rocuronium concentration after the intravenous infusion of the novel reversal agent Org 25969. Anesthesiology. 2003;99:632–7. 12960547 10.1097/00000542-200309000-00018

[18] Olkkola KT, Tammisto T. Quantifying the interaction of rocuronium (Org 9426) with etomidate, fentanyl, midazolam, propofol, thiopental, and isoflurane using closed-loop feedback control of rocuronium infusion. Anesth Analg. 1994;78:691–6. 8135387 10.1213/00000539-199404000-00013

[19] Driessen JJ, Crul JF, Vree TB, van Egmond J, Booij LH. Benzodiazepines and neuromuscular blocking drugs in patients. Acta Anaesthesiol Scand. 1986;30:642–6. 2880446 10.1111/j.1399-6576.1986.tb02492.x

[20] Yamada S, Akiyama Y, Tachibana S, Hayamizu K, Kimura Y, Hashimoto S, Yamakage M, Mikuni N. The intraoperative motor-evoked potential when propofol was changed to remimazolam during general anesthesia: a case series. J Anesth. 2023;37:154–9. 36319911 10.1007/s00540-022-03112-0

[21] Sorgenfrei IF, Norrild K, Larsen PB, Stensballe J, Ostergaard D, Prins ME, Viby-Mogensen J. Reversal of rocuronium-induced neuromuscular block by the selective relaxant binding agent sugammadex: a dose-finding and safety study. Anesthesiology. 2006;104:667–74. 16571960 10.1097/00000542-200604000-00009

[22] Shields M, Giovannelli M, Mirakhur RK, Moppett I, Adams J, Hermens Y. Org 25969 (sugammadex), a selective relaxant binding agent for antagonism of prolonged rocuronium-induced neuromuscular block. Br J Anaesth. 2006;96:36–43. 16357116 10.1093/bja/aei314

[23] Suy K, Morias K, Cammu G, Hans P, van Duijnhoven WG, Heeringa M, Demeyer I. Effective reversal of moderate rocuronium- or vecuronium-induced neuromuscular block with sugammadex, a selective relaxant binding agent. Anesthesiology. 2007;106:283–8. 17264722 10.1097/00000542-200702000-00016

[24] Nemes R, Lengyel S, Nagy G, Hampton DR, Gray M, Renew JR, Tassonyi E, Fülesdi B, Brull SJ. Ipsilateral and simultaneous comparison of responses from acceleromyography- and electromyography-based neuromuscular monitors. Anesthesiology. 2021;135:597–611. 34329371 10.1097/ALN.0000000000003896

[25] Iwasaki H, Yamamoto M, Sato H, Doshu-Kajiura A, Kitajima O, Takagi S, Luthe SK, Suzuki T. A comparison between the adductor pollicis muscle using TOF-Watch SX and the abductor digiti minimi muscle using TetraGraph in rocuronium-induced neuromuscular block: a prospective observational study. Anesth Analg. 2022;135:370–5. 35061641 10.1213/ANE.0000000000005897

[26] Pongrácz A, Szatmári S, Nemes R, Fülesdi B, Tassonyi E. Reversal of neuromuscular blockade with sugammadex at the reappearance of four twitches to train-of-four stimulation. Anesthesiology. 2013;119:36–42. 23665915 10.1097/ALN.0b013e318297ce95

